# Long Noncoding RNA Nuclear Paraspeckle Assembly Transcript 1 Promotes Progression and Angiogenesis of Esophageal Squamous Cell Carcinoma Through miR-590-3p/MDM2 Axis

**DOI:** 10.3389/fonc.2020.618930

**Published:** 2021-02-19

**Authors:** Jing Luo, Kai Xie, Xiang Gao, Yu Yao, Gaoming Wang, Chenye Shao, Xiaokun Li, Yang Xu, Binhui Ren, Liwen Hu, Yi Shen

**Affiliations:** ^1^Department of Thoracic Surgery, The Affiliated Cancer Hospital of Nanjing Medical University & Jiangsu Cancer Hospital & Jiangsu Institute of Cancer Research, Jiangsu Key Laboratory of Molecular and Translational Cancer Research, Nanjing, China; ^2^Department of Cardiothoracic Surgery, Jinling Hospital, Medical School of Nanjing University, Nanjing, China; ^3^Department of Respiratory Medicine, Nanjing Second Hospital, Nanjing, China; ^4^Department of Thoracic Surgery, Xuzhou Central Hospital, Xuzhou, China

**Keywords:** nuclear paraspeckle assembly transcript 1 (NEAT1), esophageal squamous cell carcinoma (ESCC), angiogenesis, MDM2, miR-590-3p

## Abstract

Angiogenesis has been identified as one of the hallmarks of cancer and aggravates cancer development and progression. Accumulating evidence indicated that long noncoding RNAs (lncRNAs) are powerful factors in regulating various cancer behaviors. The aim of this study is to verify the function and potential mechanisms of lncRNA NEAT1 in progression and angiogenesis of esophageal squamous cell carcinoma (ESCC). We found that NEAT1 was overexpressed in ESCC tissues and correlated with clinical characteristics of patients. Silence of NEAT1 inhibited proliferation, migration, invasion and angiogenesis of ESCC cells. High throughput sequencing and western blotting revealed that NEAT1 regulated MDM2/p53 pathway. Rescue of MDM2 restored the effect of NEAT1 on progression and angiogenesis of ESCC cells. Nude mice xenograft models further validated the role of NEAT1 *in vivo*. Importantly, NEAT1 functioned as a competing endogenous RNA for miR-590-3p to regulate MDM2 expression and miR-590-3p acted as a tumor suppressor in ESCC progression and angiogenesis. These findings suggested that NEAT1/miR-590-3p/MDM2 axis might serve as potential therapeutic targets for ESCC patients.

## Introduction

Esophageal cancer is one of the most predominant malignancies and ranks sixth in terms of cancer-related death worldwide (1). As the most predominant histological subtype of esophageal cancer, esophageal squamous cell carcinoma (ESCC) accounts for about 90% of all the esophageal cancer cases (2). Although great advances have been made in the diagnosis and therapy of ESCC, the 5-year survival rate of ESCC remains poor due to difficulties in early diagnosis and limitations of clinical therapeutic strategies (3). The molecular foundation of the initiation and progression of ESCC is still not clear. Thus, it is necessary to explore the molecular and pathogenic mechanisms of ESCC in-depth to develop novel ESCC anticancer agents.

Cancer cells require excess oxygen and nutrients for their unlimited proliferation, therefore they must recruit new blood vessels to grow beyond a critical size or metastasize to distant organ (4). Angiogenesis has been identified as the novel hallmark of cancer and is essential for sustaining the development of tumor growth and metastasis (5). The pathological angiogenesis in cancer is driven by an imbalance between pro-angiogenic and anti-angiogenic factors, which leads to replenishment of a new vessel supply (6). Tumor angiogenesis is regarded as a key target for cancer therapy and anti-angiogenic agents exhibit effective inhibition on tumor progression and metastasis (7). Hence, further research of vital molecules in the regulation of angiogenesis of ESCC might provide potential target for ESCC therapy.

Long noncoding RNAs (lncRNAs) are a class of non-protein coding transcripts over 200 nucleotides in length (8). Numerous studies have documented that lncRNAs play diverse roles in regulating multiple processes of gene expression, including transcription, post-transcription, translation, epigenetic modification, gene transportation and mRNA stability (9). Increasing evidence reveals that lncRNAs are also essential regulators in the initiation, progression and metastasis of multiple types of cancers (10). Nevertheless, the underlying function and mechanism of lncRNAs in angiogenesis of ESCC are still unclear.

LncRNA NEAT1 (nuclear paraspeckle assembly transcript 1) is retained in the nucleus and forms the core structural component of the paraspeckle sub-organelles. It is well identified that NEAT1 is aberrantly expressed in various cancers and displays carcinogenicity (11). In this research, we found that NEAT1 was up-regulated in ESCC tissues and correlated with poor clinic-pathological factors of patients. Loss of function assays demonstrated that silencing NEAT1 attenuated the progression and angiogenesis of ESCC and rescue experiments revealed that the effects of NEAT1 were dependent on MDM2/p53 axis. Mechanically, NEAT1 functioned as a ceRNA (competing endogenous RNA) to sponge miR-590-3p and further regulated MDM2 expression. In summary, our results deepen the understanding of the functional role of NEAT1 in the progression and angiogenesis of ESCC and provide novel potential targets for ESCC therapy.

## Materials and Methods

### Patient Specimens

80 paired fresh-frozen ESCC samples and adjacent normal counterparts were obtained from patients who underwent surgery between January 2015 and December 2018. All enrolled patients were diagnosed with ESCC and did not receive chemotherapy or radiotherapy before their operation. The tumor stage were defined by two experienced pathologists independently. The samples were resected within 10 min after tumor excision and stored at −80°C. The study was approved by the Ethics Committee of Jiangsu Cancer Hospital and written informed consent from all participants was obtained.

### Cell Culture and Transfection

Normal human esophageal epithelial cells (HET-1A), ESCC cell lines (ECA109, TE1, TE13, KYSE150 and KYSE140) and Human Umbilical Vein Endothelial Cells (HUVEC) were purchased from Shanghai Institutes for Biological Science (Shanghai, China). These cells were cultivated in in DMEM medium (KeyGene, Nanjing, China) supplemented with 10% fetal bovine serum (KeyGene, Nanjing, China) at 37°C in 5% CO^2^ atmosphere. SiRNAs targeting NEAT1, expression plasmids of MDM2, miR-590-3p mimics and shRNAs of NEAT1 were purchased from RiboBio, Guangzhou, China. Transfection was performed with Lipofectamine 3000 (Invitrogen, CA, USA) according to the manufacturer’s instructions. The transfected cells were incubated at 37°C for 6 h and then replaced with fresh medium. The sequences are shown in **Table S1**.

### RNA Extraction and qRT-PCR

Total RNA from tissues or cell lines was isolated with TRIzol reagent (Invitrogen, CA, USA). We synthesized cDNA by using the PrimeScript RT Master Mix (Takara, Nanjing, China). Relative RNA levels of genes were determined by RT-qPCR and ACTIN was employed as an internal control. The primers used in this study are listed in **Table S2**.

### Cell Proliferation, Migration, and Invasion Assays

Cell proliferation was assayed with CCK-8 (Beyotime, Suzhou, China) and EdU Apollo^®^488 In Vitro Imaging Kit (RiboBio, Guangzhou, China) according to the manufacturer’s instructions. The migration and invasion assays were conducted with Transwell assay inserts (8 μM PET, 24-well Millicell) and Matrigel coated inserts (BD Biosciences, Bedford, USA), respectively. These detailed experimental procedures were performed as previously described (12, 13).

### Tube Formation Assay

Precooled Matrigel (Corning, USA) was added 24-well plates and polymerized for 30 min at 37°C. HUVECs (2 × 10^4^) suspended in 200 μl of medium from each group were added to each well and incubated at 37°C for 6–12 h. The capillary-like structures were acquired under a microscope (Nikon, Tokyo, Japan). Angiogenic activity was quantified by measuring the mesh and length of the completed tubes.

### Transcriptome Sequencing

Total RNAs from ECA109 cells with NEAT1 knockdown and control cells were isolated and quantified. The concentration and purity of RNAs from different group were measured with a NanoDrop 2000 (Thermo Scientific, USA). Each sample was analyzed for quality on an Agilent 2100 Bioanalyzer (Agilent, USA). The RNAseq data analysis was performed using the Ion Proton Total RNA-Seq Kit v2 according to the protocol provided by the manufacturer (Life Technologies, USA).

### FISH (Fluorescent *In Situ* Hybridization) Assay and Subcellular Fraction Assay

FISH assay and subcellular fraction assay were performed to detect the subcellular location of lncRNA. The Ribo FISH Kit (Ribobio, Cat.R11060.7) was used to perform FISH assay. Probes for NEAT1, 18S (cytoplasmic reference) and U6 (nuclear reference) were synthesized with 3’-Cy3-modification to mark the target lncRNA and the cellular DNA was stained with DAPI. Images were photographed by fluorescence microscope. And subcellular fraction assay was conducted with the PARIS Kit (Cat. AM1921, Invitrogen, CA, USA) according to the manufacturer’s instructions.

### Pull Down Assay

HEK293T cells were cross-linked and lysed by lysis buffer containing RNase inhibitor. Biotin-labeled wide type miR-590-3p and its mutant were added to cell lysates to pull down its targets. Then, the targets were separated by magnetic support and RNAs were analyzed by qRT-PCR after purification.

### Western Blotting

Western blotting analysis was performed using standard technique. Briefly, protein extracts from cells were lysed with lysis buffer (RIPA, KeyGEN, China) containing protease inhibitors (PMSF, KeyGEN, China). Protein concentration was determined using a BCA Kit (KeyGEN, China) and total of 50 µg of protein was subjected to SDS-PAGE and transferred to 0.45 μm PVDF membrane (Millipore, China). The following primary antibodies were used: anti-β-actin (Cell Signaling Technology, 3700, 1:1000), anti-p53 (Santa Cruz Biotechnology, sc-126, 1:1,000) and anti-MDM2 (Santa Cruz Biotechnology, sc-965, 1:1,000).

### *In Vivo* Experiments

Xenograft models were used to clarify the effect of NEAT1 *in vivo*. 4-week-old BALB/c nude mice obtained from Nanjing Medical University School of Medicine’s accredited animal facility were randomly divided into two groups (sh-NC and sh-NEAT1). ECA109 cells (2 × 10^6^/100 μl) were injected subcutaneously into the right flank of these mice to establish the ESCC xenograft model. Tumors were harvested at 6 weeks after injection. Tumor volume was estimated using calipers ([length* width^2^]/2) and tumor weight was measured on the scale. The resected tumor nodules were finally paraffin embedded and subjected to immunohistochemistry (IHC) staining. Immunostaining was performed using following antibodies: anti-p53 (Santa Cruz Biotechnology, sc-126, 1:100), anti-MDM2 (Santa Cruz Biotechnology, sc-965, 1:100) and anti-CD31 (Cell Signaling Technology, 3528, 1:100).

### Dual Luciferase Reporter Assay

The wild-type (wt) or mutant (mut) of NEAT1 and 3′-UTR of MDM2 were constructed into pmirGLO vector. The construct was co-transfected with miR-NC or miR-590-3p mimics into HEK293T cells. Dual Luciferase Assay System (Promega, Madison, WI, USA) was used to detect luciferase activity after transfection for 48 h and Renilla luciferase activity was used as an internal reference.

### Statistical Analysis

Data were graphically presented with GraphPad Prism v.5.0 for Windows (GraphPad Software, CA, USA) and statistical analysis was performed using the SPSS13.0 (SPSS Inc, Chicago, IL, USA). Generally, experiments were repeated for three times, and all values were presented as means ± standard deviation (SD). The comparison of means between two groups was conducted using Student’s t test. P < 0.05 was considered significant.

## Results

### Nuclear Paraspeckle Assembly Transcript 1 Was Highly Expressed in Esophageal Squamous Cell Carcinoma Tissues and Linked to Poor Clinic-Pathological Factors

**Figure 1:**
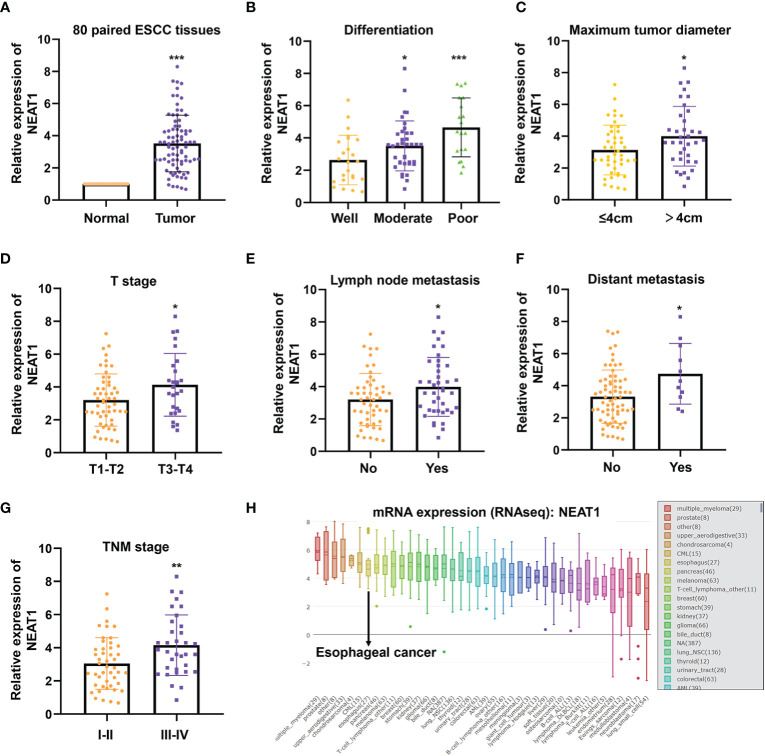
NEAT1 was up-regulated in ESCC and correlated with clinical features. **(A)** The expression of NEAT1 in 80 paired ESCC tissues was detected by qRT-PCR and results indicated that NEAT1 was significantly upregulated in ESCC tissues compared with adjacent normal tissues (p < 0.001). **(B)** Tumor tissues of moderate and poor differentiation bore higher expression of NEAT1 relative to those of well differentiation (p < 0.05; p < 0.001). **(C)** NEAT1 was more highly expressed in tumor tissues with larger size (p < 0.05). **(D)** The upregulaticlinicalon of NEAT1 correlated with more advanced T stages of patients (p < 0.05). **(E, F)**. Patients with lymph node or distant metastasis exerted higher expression of NEAT1 (p < 0.05; p < 0.05). **(G)** NEAT1 showed higher expression in patients with later TNM stage (p < 0.01). **(H)** The mRNA levels of NEAT1 in different cancer cell lines were presented by CCLE. *P < 0.05, **P < 0.01, ***P < 0.001, n.s., no significance.

QRT-PCR was performed to verify the expression of NEAT1 in 80 paired ESCC and adjacent normal tissues, and we found that NEAT1 was significantly up-regulated in ESCC tissues (**Figure 1A**). Furtherly we analyzed the expression of NEAT1 in tumor tissues with different clinical characteristics. Results suggested that the upregulation of NEAT1 correlated with poorer differentiation (**Figure 1B**), larger tumor size (**Figure 1C**), later T stage (**Figure 1D**), lymph node metastasis (**Figure 1E**), distant metastasis (**Figure 1F**) and later TNM stage (**Figure 1G**). And the mRNA levels of NEAT1 in multiple types of cancer cells were sought by CCLE database. Consistently, NEAT1 exerted a relative high level in esophageal cancer cells (**Figure 1H**). These data implied that NEAT1 was remarkably upregulated in ESCC tissues and correlated with poor clinic-pathological factors of patients.

### Knockdown of Nuclear Paraspeckle Assembly Transcript 1 Suppressed Progression and Angiogenesis of Esophageal Squamous Cell Carcinoma *In Vitro*

**Figure 2:**
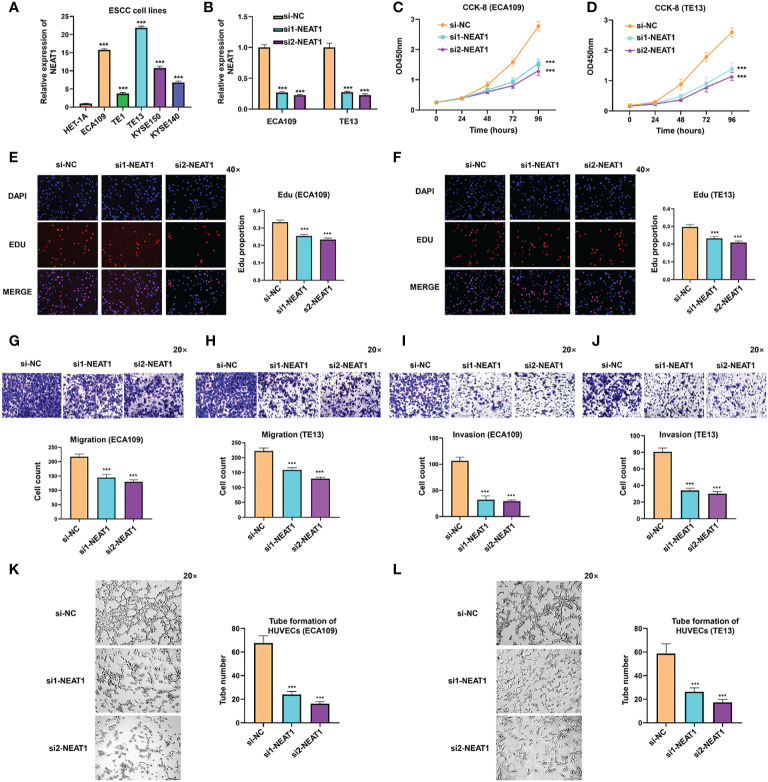
Knockdown of NEAT1 suppressed proliferation, migration and invasion of ESCC cells. **(A)** The expression of NEAT1 in common ESCC cell lines was verified by qRT-PCR and results showed that NEAT1 exhibited generally high levels in ESCC cell lines compared with human esophageal epithelial cell (HET-1A). **(B)** Two siRNAs were constructed and transfected into ECA109 and TE13 cells, and both siRNAs effectively knocked down the expression of NEAT1. **(C–F)** CCK-8 and Edu assays were performed to detect the proliferation rate of ESCC cells after knockdown of NEAT1 and it revealed that silencing NEAT1 significantly inhibited proliferation of ECA109 and TE13 cells. **(G–J)** Transwell and Matrigel assays suggested that knockdown of NEAT1 inhibited ECA109 and TE13 cells to migrate and invade. **(K, L)** HUVECs were seeded onto a plate precoated with Matrigel and incubated with conditioned medium from ECA109 or TE13 cells treated with silencing NEAT1 or control. Results showed that medium from cells of silencing NEAT1 significantly inhibited the tube formation of HUVECs relative to control. *P < 0.05, **P < 0.01, ***P < 0.001, n.s., no significance.

To further explore the biological function of NEAT1 in ESCC, we firstly examined the expression of NEAT1 in some common ESCC cell lines. Compared with human esophageal epithelial cell (HET-1A), NEAT1 was at relatively high levels in five ESCC cell lines (ECA109, TE1, TE13, KYSE150, and KYSE140) (**Figure 2A**). And we transfected two specific siRNAs into ECA109 and TE13 cells to knockdown the expression of NEAT1. Confirmed by qRT-PCR, both siRNAs effectively downregulated the expression of NEAT1 in ESCC cells (**Figure 2B**). CCK-8 and Edu assays revealed that the proliferation rate of ECA109 and TE13 cells was evidently reduced after knockdown of NEAT1 (**Figures 2C–F**). Transwell and Matrigel assays indicated that knockdown of NEAT1 markedly decreased the migration and invasion ability of ECA109 and TE13 cells (**Figures 2G–J**). Moreover, Matrigel capillary tube formation assay was utilized to assess the effect of NEAT1 in angiogenesis. Medium from ECA109 or TE13 cells with NEAT1 knockdown dramatically inhibited tube sprout of HUVECs compared with negative control (**Figures 2K, L**). These results uncovered the role of NEAT1 in promoting progression and angiogenesis of ESCC and it is of great significance to research its mechanism of action.

### Knockdown of Nuclear Paraspeckle Assembly Transcript 1 Regulated MDM2-p53 Signaling Pathway

**Figure 3:**
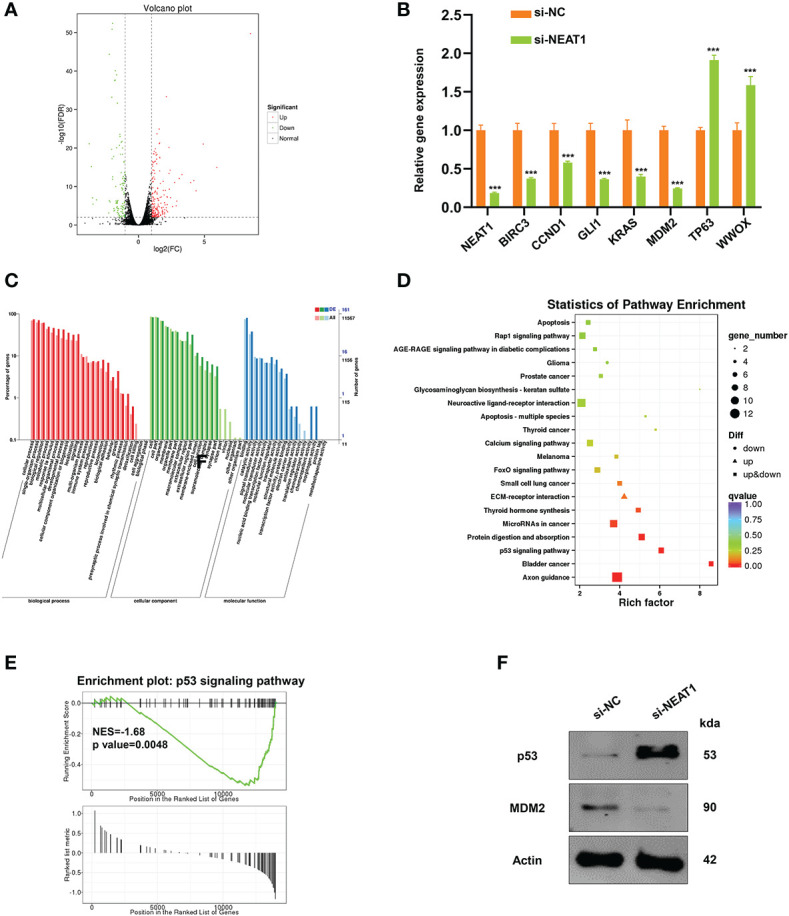
NEAT1 regulated MDM2-p53 signaling pathway in ESCC cells. **(A)** Volcano plot showed the most differentially expressed genes (fold-change> 2.0) between NEAT1-silenced ECA109 cells and control group, of which 212 genes were upregulated and 114 genes were downregulated. **(B)** Verified by qRT-PCR, knockdown of NEAT1 reduced the mRNA expression of some oncogenes (BIRC3, CCND1, GLI1, KRAS, MDM2) and upregulated several tumor suppressor genes (TP63, WWOX). **(C)** GO annotation showed that the differentially expressed genes by silencing NEAT1 participated in multiple biological process and molecular function. **(D, E)** Pathway enrichment analysis and GSEA (gene-set enrichment analysis) indicated that genes in response to NEAT1 knockdown were significantly related to p53 signaling pathway. **(F)** Knockdown of NEAT1 regulated the protein level of MDM2 and p53 in ECA109 cells. *P < 0.05, **P < 0.01, ***P < 0.001, n.s., no significance.

RNA transcriptome sequencing was performed in ECA109 cells with NEAT1 silencing or negative control to ascertain the mechanism of action of NEAT1-driven oncogenesis of ESCC. Totally the expression of 326 genes were changed (fold change ≥2) as a consequence of NEAT1 knockdown, of which 212 genes were upregulated and 114 genes were downregulated (**Figure 3A**). We performed qRT-PCR in ECA109 cells and results confirmed that knockdown of NEAT1 altered the mRNA expression of several oncogenes (BIRC3, CCND1, GLI1, KRAS, and MDM2) and tumor suppressor genes (TP63 and WWOX) (**Figure 3B**). GO annotation showed that those differentially expressed genes (DEG) in RNA transcriptome sequencing participated in multiple biological process and molecular function (**Figure 3C**). While pathway enrichment analysis and GSEA (gene-set enrichment analysis) prompted that NEAT1 was involved in the regulation of p53 signaling pathway (**Figures 3D, E**). As MDM2 encodes an E3 ubiquitin ligase and promotes tumor formation by targeting tumor suppressor proteins p53 for proteasomal degradation (14), we speculated that NEAT1 might regulate the p53 signaling pathway through MDM2. And western blotting proved that knockdown of NEAT1 altered the protein level of MDM2 and p53 in ECA109 cells (**Figure 3F**). These results implied that NEAT1 might exerted its role in cancer progression and angiogenesis through MDM2-p53 signaling pathway.

### MDM2 Restored the Promoting Effect of Nuclear Paraspeckle Assembly Transcript 1 on Tumor Progression and Angiogenesis in Esophageal Squamous Cell Carcinoma Cell

**Figure 4:**
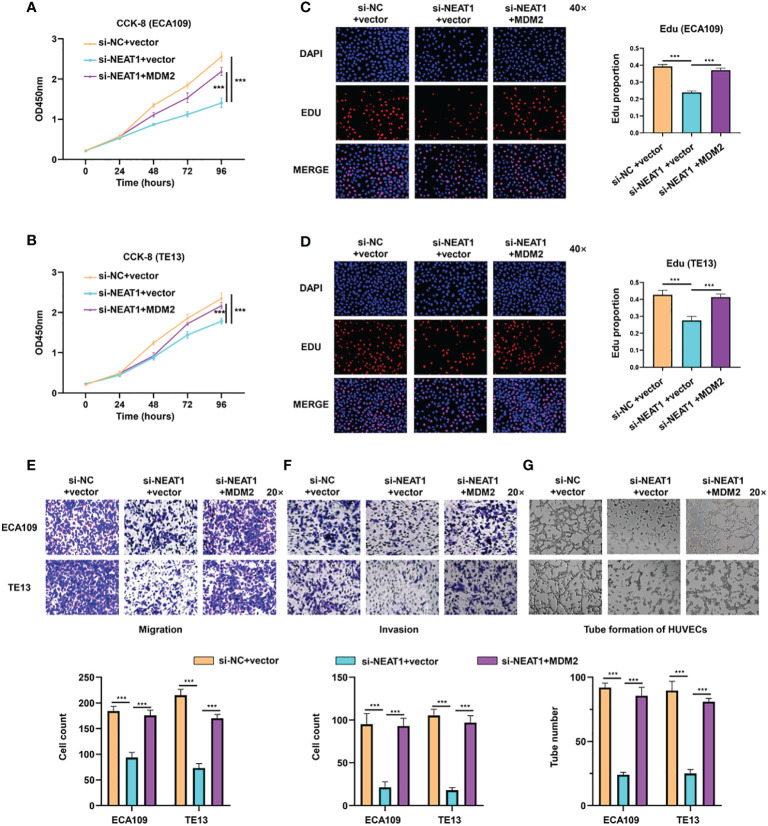
NEAT1 regulated progression and angiogenesis of ESCC cells *via* partly modulating MDM2 expression. **(A–D)** Validated by CCK8 assay and Edu assay, ectopic expression of MDM2 restored the inhibitory effect of si-NEAT1 on proliferation in ECA109 and TE13 cells. **(E, F)** The migration and invasion ability of ESCC cells inhibited by si-NEAT1 could be recovered by ectopic expression of MDM2 in Transwell and Matrigel assays. **(G)** Tube formation assays of HUVEC cells were performed and it indicated that the angiogenesis inhibition of si-NEAT1 could be rescued by MDM2. *P < 0.05, **P < 0.01, ***P < 0.001, n.s: no significance.

Rescue experiments were performed in ESCC cells to evaluate whether the function of NEAT1 was relied on MDM2. Briefly, expression plasmids of MDM2 were transfected into si-NETA1-treated ESCC cells. CCK8 and Edu assays showed that ectopic expression of MDM2 could rescue the negative effect of silencing NEAT1 on cell proliferation in ECA109 and TE13 cells (**Figures 4A–D**). Likewise, Transwell and Matrigel assays revealed that the migration and invasion of ESCC cells inhibited by knockdown of NEAT1 could be recovered through overexpression of MDM2 (**Figures 4E, F**). Moreover, introduction of MDM2 expression plasmid restored the angiogenesis inhibition of si-NEAT1 (**Figure 4G**). Overall, we found that NEAT1 exerted its role in progression and angiogenesis of ESCC cells *via* partly regulating MDM2 expression.

### Nuclear Paraspeckle Assembly Transcript 1 Promoted Progression of Esophageal Squamous Cell Carcinoma *In Vivo*

**Figure 5:**
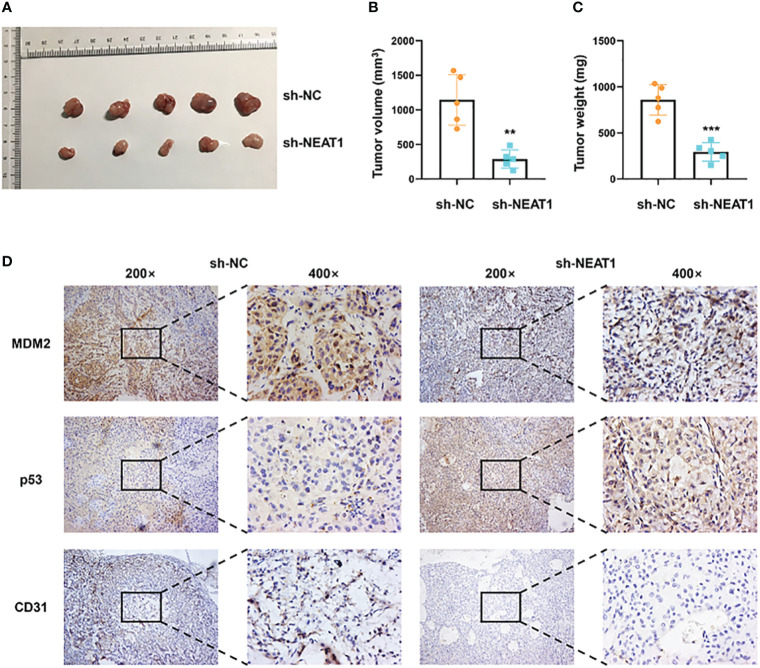
Knockdown of NEAT1 suppressed cancer progression *in vivo*. **(A)** Tumor nodules of sh-NC and sh-NEAT1 groups were harvested. **(B, C)** Downregulation of NEAT1 suppressed tumor growth of ESCC *in vivo*, as indicated by tumor volume and weight. **(D)** The expression of MDM2, p53 and CD31 in tumor were measured by IHC staining and results indicated that NEAT1 regulated MDM2-p53 axis and angiogenesis of ESCC *in vivo*. *P < 0.05, **P < 0.01, ***P < 0.001, n.s, no significance.

The xenograft models were utilized to explore the function of NEAT1 *in vivo*. ECA109 cells transfected with shRNA-NEAT1 or shRNA-control were injected into axilla subcutaneously of mice and tumor nodules were harvested at 6 weeks after injection (**Figure 5A**). Tumor volume and weight were measured and results showed that downregulation of NEAT1 in ECA109 cells significantly reduced the xenograft tumor growth (**Figures 5B, C**). IHC staining of harvested tumors implied that knockdown of NEAT1 reduced the expression of MDM2 and CD31 (marker of angiogenesis) and upregulated the expression of p53 *in vivo* (**Figure 5D**). These results demonstrated that knockdown of NEAT1 inhibited cancer development and angiogenesis of ESCC *in vivo*.

### Nuclear Paraspeckle Assembly Transcript 1 Functioned as a Competing Endogenous RNA for miR-590-3p to Regulate MDM2 Expression

**Figure 6:**
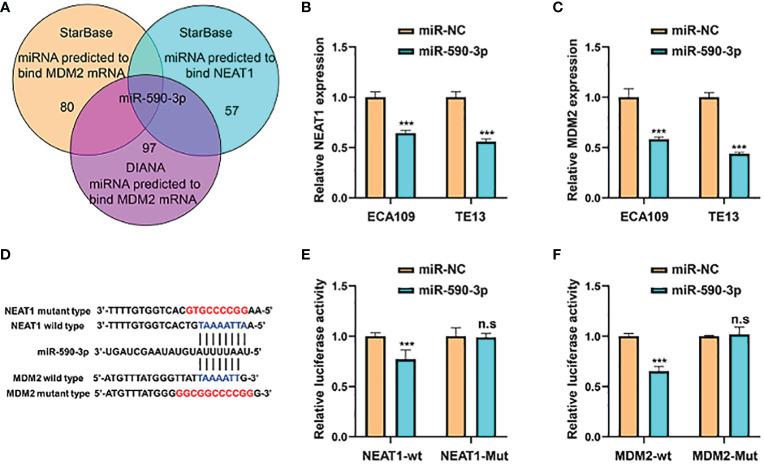
NEAT1 sponged miR-590-3p to regulate the expression of MDM2. **(A)** Venn diagram for screening miRNAs that potentially target both NEAT1 and MDM2. **(B, C)** Relative to negative control miR-NC, miR-590-3p decreased the mRNA expression of NEAT1 and MDM2 in ECA109 and TE13 cells. **(D)** Schematic of wild-type and mutant constructs of NEAT1 and MDM2. **(E, F)** MiRNA mimics and luciferase vectors were co-transfected into HEK293T cells and it revealed that miR-590-3p reduced the luciferase activity of wild-type NEAT1 and MDM2 reporters, whereas no significant effect was observed when the putative binding site was mutated. *P < 0.05, **P < 0.01, ***P < 0.001, n.s, no significance.

Competing endogenous RNAs (ceRNAs) are transcripts that can regulate each other at post-transcription level by competing for shared miRNAs. Accumulating evidence has indicated that numerous lncRNAs exerted biological roles in a ceRNA-mediated mechanism (15). Through multiple bioinformatics databases prediction, we found that NEAT1 might regulate the expression of MDM2 by sponging miR-590-3p (**Figure 6A**). Treatment of miR-590-3p mimics significantly inhibited mRNA levels of NEAT1 and MDM2 in ECA109 and TE13 cells (**Figures 6B, C**). Wild-type and mutant-type luciferase vectors were constructed according to the putative binding sites of miR-590-3p with MDM2 and NEAT1 (**Figure 6D**) and dual luciferase assays were conducted in HEK293T cells to validate the binding. Results showed that miR-590-3p inhibited the luciferase reporter activity of wild-type NEAT1 and MDM2, but not of the mutant type (**Figures 6E, F**). And in pull down assay, NEAT1 and MDM2 were detected in the fragments pulled down by wild-type bio-miR-590-3p (WT), but not the mutant type (MUT) (**Figure S1**). These data suggested that NEAT1 regulated the expression of MDM2 *via* sponging miR-590-3p.

### MiR-590-3p Inhibited Progression and Angiogenesis of Esophageal Squamous Cell Carcinoma Cells

**Figure 7:**
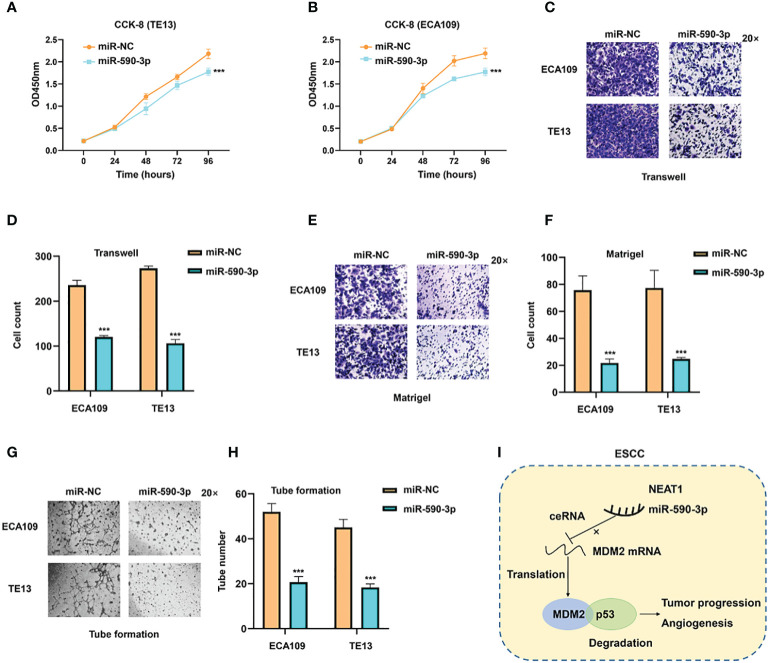
MiR-590-3p was a suppressor of cancer progression and angiogenesis in ESCC cells. **(A, B)** CCK8 assays implied that miR-590-3p inhibited proliferation of ECA109 and TE13 cells. **(C–F)** Transwell and Matrigel assays indicated that miR-590-3p inhibited migration and invasion of ECA109 and TE13 cells. **(G, H)**. Indicated by tuber formation assays, miR-590-3p suppressed angiogenesis of ECA109 and TE13 cells. **(I)** The graphic illustration of function and mechanisms of NEAT1 in regulating progression and angiogenesis of ESC **(C)** *P < 0.05, **P < 0.01, ***P < 0.001, n.s, no significance.

As miR-590-3p was a sponge for NEAT1 and regulated its expression, we furtherly investigated the role of miR-590-3p on cancer progression and angiogenesis in ESCC. Validated by CCK8 assay, miR-590-3p inhibited tumor proliferation of ECA109 and TE13 cells (**Figures 7A, B**). Transwell and Matrigel assays indicated that miR-590-3p suppressed ESCC cells to migrate and invade (**Figures 7C, F**). Likewise, tube formation assays of HUVEC cells were performed and results showed that miR-590-3p reduced angiogenesis of ECA109 and TE13 cells (**Figures 7G, H**). These findings suggested that miR-590-3p functioned as a suppressor of cancer progression and angiogenesis in ESCC cells.

## Discussion

At present, ESCC is one of the most common malignancies worldwide, especially in China (16). The poor prognosis of ESCC is principally due to indistinct mechanism of tumor progression and lack of treatment methods for patients of late stage, therefore developing novel treatment targets for ESCC is an indispensable requisite. In this study, we identified that lncRNA NEAT1 was abnormally highly expressed in ESCC tissues and correlated with clinical characteristics of patients. Knockdown of NEAT1 inhibited ESCC progression and angiogenesis *in vitro* and *in vivo*, partially through regulating MDM2-p53 signaling pathway. Furthermore, NEAT1 modulated the expression of MDM2 *via* functioning as a ceRNA to sponge miR-590-3p and miR-590-3p was validated as a tumor suppressor in ESCC progression and angiogenesis. Our results uncovered a novel mechanism by which NEAT1 promotes progression and angiogenesis of ESCC and might provide useful diagnostic and treatment targets for ESCC patients.

Nuclear paraspeckle assembly transcript 1 (NEAT1) was reported to be widely expressed in a variety of mammalian cell types (17). And it was well identified that NEAT1 was involved in multiple physiological and pathological processes, including transcriptional regulation (18), alternative splicing (19), virus infection (20), neuronal activity (21), tumor microenvironment regulation (22) and immune response (23). Accumulating evidences suggested that NEAT1 plays crucial roles in carcinogenesis of multiple types of cancers, including breast cancer (24), colorectal cancer (25), nasopharyngeal cancer (26) and gastric cancer (27). And it has been documented that upregulation of NEAT1 correlates with poor prognosis of ESCC (28) and NEAT1 regulates cell viability and invasion in ESCC through the miR-129/CTBP2 Axis (29). We discovered that NEAT1 exerted oncogenic role in progression and angiogenesis of ESCC. In addition to extending the function of NEAT1 in tumor development, our study deepens the understanding of the impact of lncRNAs in the angiogenesis of cancer.

Cancer cells induce the formation of new blood vessels for their survival and angiogenesis has been implied as a new hallmark of cancer, which is an important event for solid tumor growth and dissemination. Effective inhibition of tumor angiogenesis has been identified to be a valid target for the treatment of many solid tumors and in particular their metastases (30). Recently multiple lncRNAs are reported to function in angiogenesis of various cancers. DANCER (Differentiation antagonizing non-protein coding RNA), a newly identified oncogenic lncRNA found in many kinds of cancers, played a promotional role in tumor angiogenesis in ovarian cancer through regulation of miR-145/VEGF axis (31). In colorectal cancer, lncRNA GAS5 inhibited the activation of the Wnt/β-catenin signaling pathway, thereby suppressing the angiogenesis, invasion, and metastasis (32). Previous study revealed that NEAT1 facilitated survival and angiogenesis in oxygen-glucose deprivation (OGD)-induced brain microvascular endothelial cells (BMECs) *via* targeting miR-377 (33). Our study firstly demonstrated that NEAT1 promoted angiogenesis in ESCC *via* modulating MDM2/p53 pathway. MDM2 is a proto-oncogene and promotes tumor formation by targeting tumor suppressor proteins p53 for proteasomal degradation (34). Researches have reported that MDM2 overexpression modulates the angiogenesis-related gene expression profile of prostate cancer cells (35) and MDM2 inhibitors exerted anti- angiogenesis effects in human breast cancer and neuroblastoma (36, 37). Moreover, the tumor suppressor p53 has been verified to play crucial roles in cancer angiogenesis through a cluster of complex mechanisms (38–40). Our data identified the pro-angiogenic role of NEAT1 in ESCC, of which the function could be rescued by altering the expression of MDM2. As a target of NEAT1, miR-590-3p exerted adverse anti-angiogenic role. These data indicated that the NEAT1/miR-590-3p/MDM2 axis played vital roles in the angiogenesis of ESCC and our results provided potential target for anti-cancer therapy in ESCC.

The most common mode that lncRNAs works is to function as ceRNAs to sponge certain microRNAs hence relieving repression of target mRNAs at a post-transcriptional level (41). We further found that NEAT1 sponged miR-590-3p to regulate the expression of MDM2. Plenty of studies have demonstrated that miR-590-3p was aberrantly expressed in multiple types of cancers and acted as a tumor suppressor, including osteosarcoma (42), breast cancer (43), hepatocellular carcinoma (44), and glioblastoma (45). In addition, several studies uncovered the role of miR-590-3p in angiogenesis. Inhibition of miR-590-3p promoted interleukin-18 expression and angiogenesis of endothelial progenitor cells (46), and NORAD/miR−590−3p axis was a novel regulatory pathway in the angiogenic mechanisms in HUVECs under hypoxia (47). The enhancing effect of miR-590-3p in angiogenesis of ESCC was also validated in this study. Although FISH assay and subcellular fraction assay in ECA109 and TE13 cells indicated that NEAT1 mainly localized in nucleus (ECA109, 72.73% in nucleus; TE13, 73.94% in nucleus) (**Figure S2**), our data suggested that NEAT1 functioned through sponging miR-590-3p to regulate MDM2, or at least, partially through this ceRNA network.

In summary, our results highlighted that the upregulation of NEAT1 in ESCC promoted progression and angiogenesis *in vitro* and *in vivo*. And NEAT1 exerted its biological role through sponging miR-590-3p to regulate MDM2/p53 axis (**Figure 7I**). These data provided potential therapeutic targets for ESCC treatment.

## Data Availability Statement

The datasets presented in this study can be found in online repositories. The names of the repository/repositories and accession number(s) can be found below: SRA, PRJNA670298.

## Ethics Statement

The studies involving human participants were reviewed and approved by the Ethics Committee of the Nanjing Medical University Affiliated Cancer Hospital. The patients/participants provided their written informed consent to participate in this study. The animal study was reviewed and approved by Nanjing Medical University Animal Care Committee.

## Author Contributions

YS, BR, and LH designed and supervised the study. JL, KX, and XG performed most experiments and wrote the manuscript. YY, GW, and CS helped to perform parts of the experiments. Xl and YX analyzed the data and revised the article. All authors contributed to the article and approved the submitted version.

## Funding

This research was supported by the Basic Research Program of Jiangsu Province (BK20160606), National Natural Science Foundation for Youth of China (nos. 81902354 and 81702444), Natural Science Foundation of Jiangsu Province (BK20181239), National Natural Science Foundation of China (81672869), Jiangsu Provincial Medical Outstanding Talent (Lin Xu), Jiangsu Provincial Medical Youth Talent (BR, QNRC2016657), the Talents Program of Jiangsu Cancer Hospital YC201814 and the “333” Talent Project: BRA2019325.

## Conflict of Interest

The authors declare that the research was conducted in the absence of any commercial or financial relationships that could be construed as a potential conflict of interest.
