# Supplementary material for: Long Noncoding RNA Nuclear Paraspeckle Assembly Transcript 1 Promotes Progression and Angiogenesis of Esophageal Squamous Cell Carcinoma Through miR-590-3p/MDM2 Axis
Source: Front Oncol. 2021 Feb 19;10:618930. doi: 10.3389/fonc.2020.618930 (PMC7933463; doi:10.3389/fonc.2020.618930)
Supplement: Supplementary file 1 [file DataSheet_1.pdf]

FigureS1: Pull down assays revealed that miR-590-3p bound with NEAT1 and MDM2 mRNA. A-B. Expression levels of NEAT1 and MDM2 in samples that were pulled down by biotinylated miR-590-3p were detected by qRT-PCR.

FigureS2: FISH and subcellular fraction assay indicated that NEAT1 mainly localized in nucleus. A-B. Results of FISH and subcellular fraction assay in ECA109 cells. C-D. Results of FISH and subcellular fraction assay in TE13 cells.
