# Supplementary figures and images for: Long Noncoding RNA Nuclear Paraspeckle Assembly Transcript 1 Promotes Progression and Angiogenesis of Esophageal Squamous Cell Carcinoma Through miR-590-3p/MDM2 Axis
Source: Front Oncol. 2021 Feb 19;10:618930. doi: 10.3389/fonc.2020.618930 (PMC7933463; doi:10.3389/fonc.2020.618930)

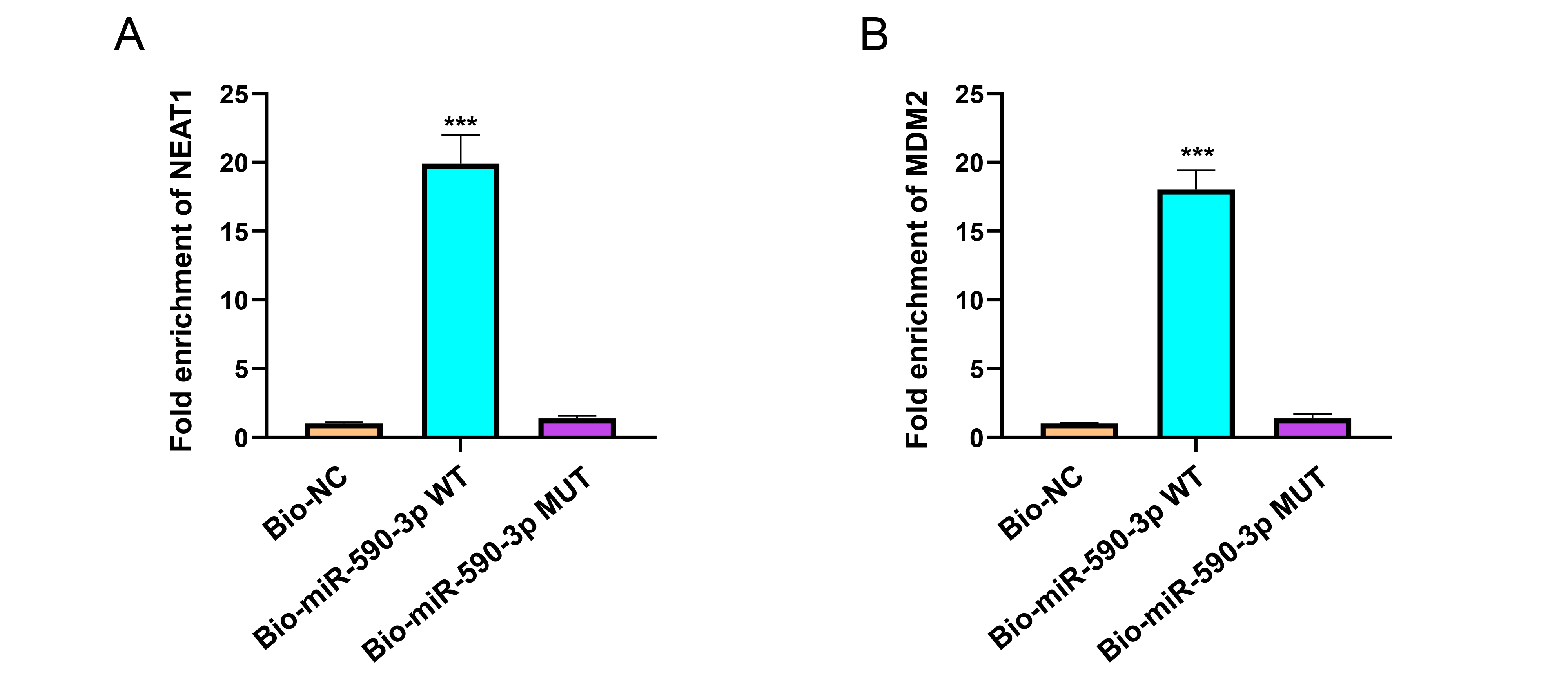

Supplement: Supplementary file 2 [file Image_1.tif]

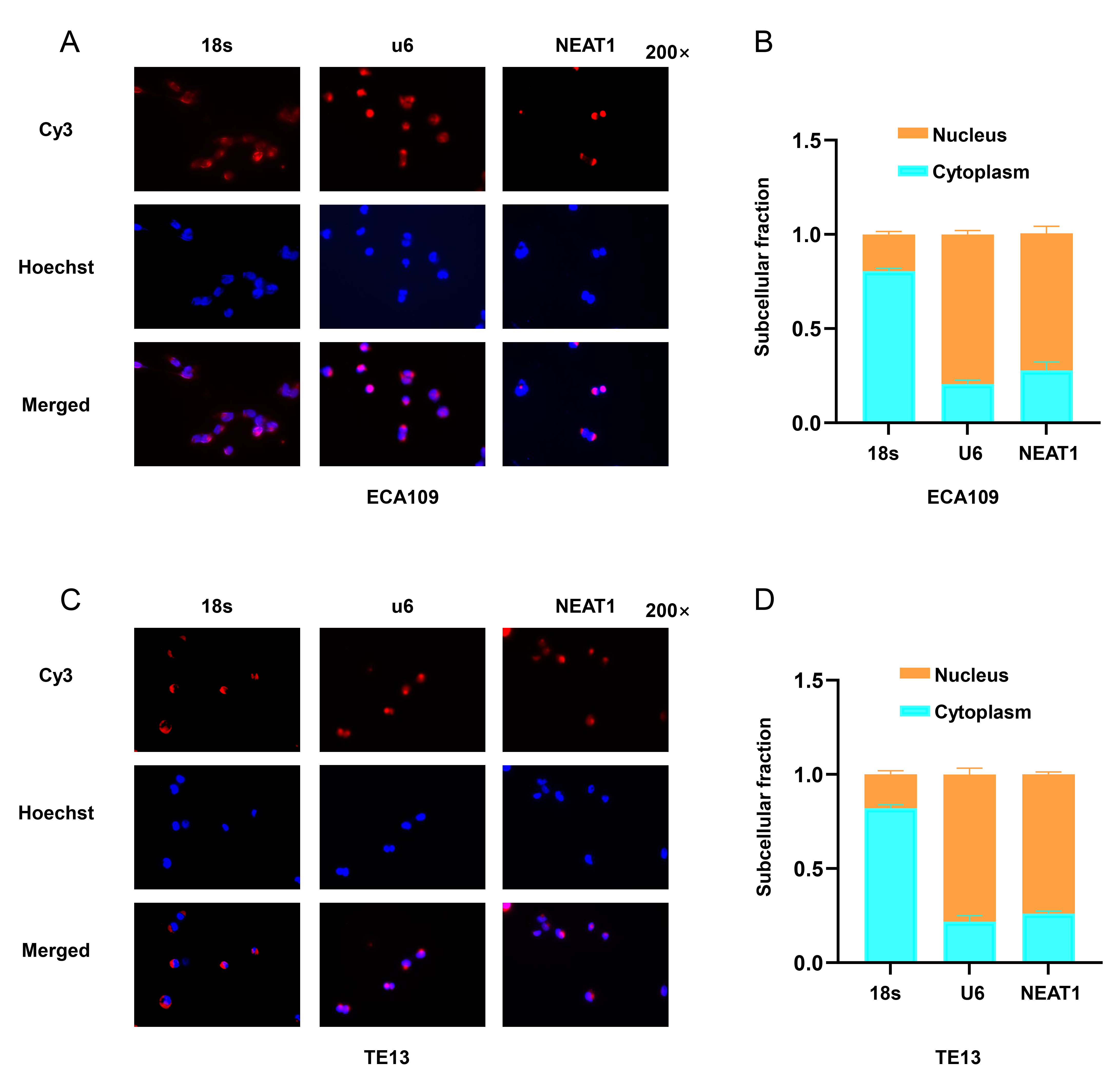

Supplement: Supplementary file 3 [file Image_2.tif]
